# 165. Biofire pneumonia panel use in severe pneumonia and antibiotic treatment in COVID-19 patients

**DOI:** 10.1093/ofid/ofac492.243

**Published:** 2022-12-15

**Authors:** Erica J Stohs, Maureen McElligott, Hannah M Creager, Paul D Fey, Trevor C Van Schooneveld

**Affiliations:** University of Nebraska Medical Center, Omaha, Nebraska; UNMC Pulmonary and Critical Care Medicine, Omaha, Nebraska; University of Pittsburgh Medical Center, Pittsburgh, Pennsylvania; Univeristy of Nebraska Medical Center, Omaha, Nebraska; University of Nebraska Medical Center, Omaha, Nebraska

## Abstract

**Background:**

Hospitalized COVID-19 patients with severe pneumonia are commonly treated for secondary bacterial pneumonia. The Biofire pneumonia panel, a rapid molecular diagnostic tool with 18 bacterial, 8 viral and 7 resistance gene targets, was made available to critical care and infectious disease clinicians in May 2020 at our institution. We sought to describe its utilization and influence on antibiotic use in patients hospitalized with COVID-19 lower respiratory tract infection (LRTI).

**Methods:**

Eligible patients with COVID-19 LRTI (positive PCR test and abnormal chest imaging) had sputum or bronchoalveolar lavage pneumonia panel (PNP) paired with a respiratory tract culture between May 4 and Dec. 8, 2020, were included. Demographics, comorbidities, clinical data including microbiologic testing, PNP indication(s), and antibiotic use after testing were abstracted through chart review. Descriptive statistics were utilized.

**Results:**

Characteristics of 133 patients are provided in Table 1. Median age was 61 years, 93 (70%) were male, 93 (70%) were mechanically ventilated, and 68 (51%) died within 30 days on PNP testing. PNP results, including culture results are listed in Table 2. No target was identified in 63 (47%) patients. *Staphylococcus aureus* was the most common bacterial isolate identified (MSSA in 32 [24%], MRSA in 8 [6%]) with culture growth in 21 specimens. More than 1 target was identified in 29 patients (22%). Empiric antibiotics and subsequent modifications within 24h hours of pneumonia panel are provided in Table 3. Vancomycin and cefepime were most frequently prescribed. Antibiotic modifications were made in 71/133 patients. Cessation of the anti-MRSA agent occurred in 39/72 (54%) of eligible patients and the anti-Pseudomonal agent in 21/78 (27%).

**Figure:**
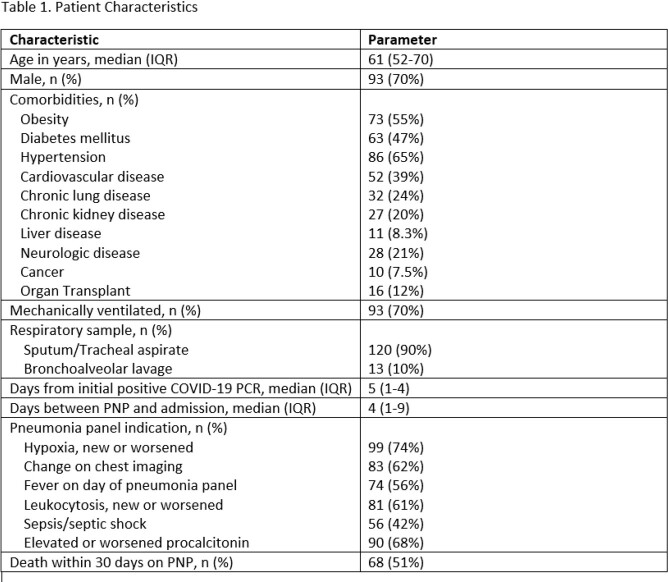

**Figure:**
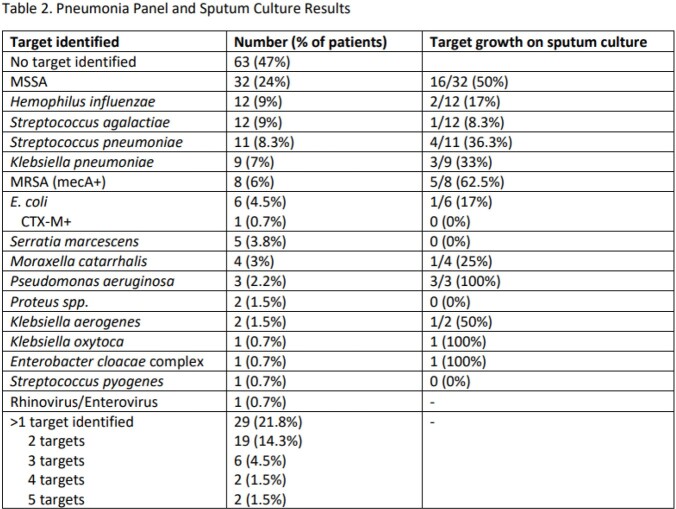

**Figure:**
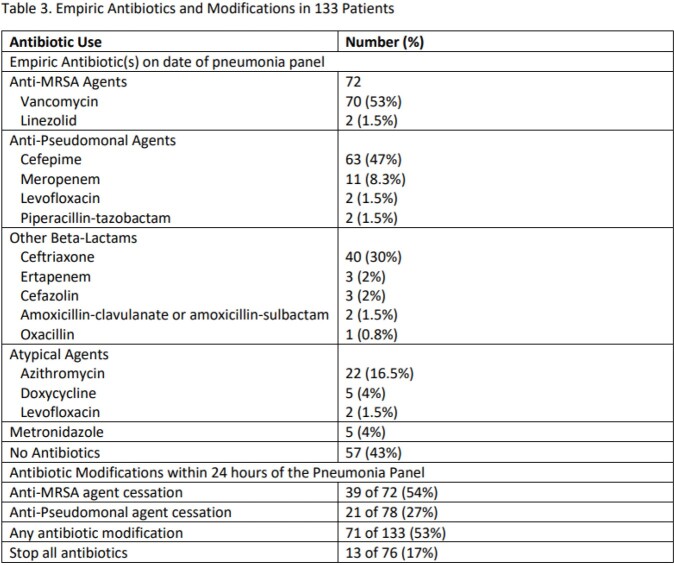

**Conclusion:**

The PNP is a useful tool to evaluate secondary bacterial pneumonia in critically ill COVID-19 patients and may assist clinicians and antimicrobial stewardship programs in expedited antibiotic discontinuation or de-escalation particularly where rates of secondary bacterial infection are low, such as COVID-19 LRTI.

**Disclosures:**

**Erica J. Stohs, MD, MPH**, bioMerieux: Grant/Research Support **Paul D. Fey, PhD**, BioFire: Advisor/Consultant|BioFire: Grant/Research Support|Merck: Grant/Research Support **Trevor C. Van Schooneveld, MD**, bioMerieux: Advisor/Consultant|bioMerieux: Grant/Research Support|Insmed: Grant/Research Support|Merck: Grant/Research Support|Thermo-Fischer: Advisor/Consultant.

